# Winnie Mice: A Chronic and Progressive Model of Ulcerative Colitis

**DOI:** 10.1093/ibd/izaf006

**Published:** 2025-02-06

**Authors:** Marcello Chieppa, Stefania De Santis, Giulio Verna

**Affiliations:** Department of Experimental Medicine, University of Salento, 73100 Lecce, Italy; Department of Pathology, Case Western Reserve University School of Medicine, Cleveland, OH 44106, USA; Digestive Health Research Institute, Case Western Reserve University School of Medicine, Cleveland, OH 44106, USA

**Keywords:** animal models of IBD, chronic intestinal inflammation, *Muc2* mutation, ulcerative colitis

## Abstract

Recent trends show a continuous worldwide rise in the incidence of ulcerative colitis (UC), leading to increased interest in its etiology and pathogenesis, which is currently unknown. To gain a better mechanistic understanding of this disease, many mouse models have been developed over the last several years, with variations of dextran sodium sulfate administration representing the most widely employed. The Winnie mouse strain was created through elicited random mutations in *Muc2*, resulting in a progressive, chronic intestinal inflammation localized to the colon that worsens over time. Moreover, Winnie mice display immunologic and microbiota features that are similar to those that can be found in UC patients. Phenotypically, the presence, albeit rare, of rectal prolapse and other complications impacting quality of life can be observed in Winnie mice, as well as extraintestinal manifestations that are often associated with UC. While Winnie mice are currently less studied compared to other more established models of colitis, much has been discovered in the initial years of its use as a UC-like model. In summary, the use of Winnie mice adds to the growing armamentarium that is required to develop precision-based medicine for its future application in treating complex multifactorial diseases, such as UC.

**Key Messages**

**• What Is Already Known?**

Dextran sodium sulfate is an established, chemically induced colitis model that is widely used and easy to perform, but has limitations in regard to recapitulating several important features of ulcerative colitis (UC).

**• What Is New Here?**

Winnie mice represent a relatively novel model of UC-like colitis that develops chronic intestinal inflammation that worsens over time and displays characteristics resembling the human condition.

**• How Can This Study Help Patient Care?**

Use of Winnie mice as a pre-clinical model is ideal to investigate the natural course of UC—prior to the onset, during active inflammation, and when chronic colitis is established and can be used to design more targeted, precision-based therapies for patients suffering from this devastating condition.

## Introduction

Ulcerative colitis (UC), is among the most frequent chronic inflammatory syndromes, characterized by genetic and environmental components, which occur within Westernized lifestyle/environment(s). In North America and Europe, the incidence of UC is estimated to be 10-20 cases per 100 000 persons per year.^1^ The genetic component of the disease is evident, considering that the risk of developing UC is estimated to be 4 times higher in first-degree relatives of UC patients compared to that in the general population. Importantly, there is not a single mutation associated with UC, but rather a variety of genetic variants involved in a complex cascade of events leading to similar symptoms, collectively recognized as UC. Among the numerous genetic susceptibility loci identified by genome-wide association study (GWAS) are mutations leading to defects in the synthesis of mucin proteins, which have been recently described.^2^ The importance of the intestinal mucus layer is not surprising, particularly considering the role of the inner mucus layer in creating an impenetrable wall between luminal bacteria and intestinal epithelial cells.^3,4^ Indeed, constant exposure to intestinal bacteria may lead to aberrant immune cell activation, which can result in a self-renewing vicious circle leading to the pathogenic manifestations of UC, whereas germ-free (GF) animal models are usually protected. Drugs that are able to suppress the inflammatory response or limit immune cell recruitment to the gut mucosa are commonly used in clinical practice with satisfactory results. Nonetheless, there is still a percentage of patients that fail to respond or lose the ability to respond over time.^5^

In order to provide meaningful insights into the histopathologic and morphologic changes of the intestinal tract related to UC pathogenesis and treatment of patients with UC, a variety of murine models have been developed; these models can be broadly categorized as: chemically induced, spontaneous, genetically modified, and adoptive transfer models.^6^ While results obtained from these models over the last several years have uncovered important, fundamental concepts in the pathogenesis of inflammatory bowel disease (IBD), a faithful representation concerning the complexity of both the clinical and histopathological characteristics of human disease has been difficult to achieve. Notably, a relatively novel, and under-utilized, model of UC, that is, Winnie mouse strain, is emerging as an ideal model to study UC that better resembles the pathology and progression of patients living with UC. This review aims to fully characterize Winnie mice, emphasizing the high fidelity with the human condition and its potential use as a reliable, pre-clinical tool for investigating and developing novel treatment modalities for patients suffering this devastating disease.

## Commonly Used Mouse Models of Colitis

Currently, the most frequently used UC models rely on the administration of dextran sodium sulfate (DSS) in drinking water for various periods of time.^7^ The resulting colitis in this model is not directly induced by the sulfated polysaccharide, but rather by selective action of the chemical toxin on the epithelium, resulting in epithelial cell injury and interruption of barrier integrity. The ensuing epithelial damage supports the entry of luminal bacteria and associated antigens into the gut mucosa, with consequent infiltration of inflammatory cells into the underlying tissues. Several factors influence the effectiveness of this model, including the frequency and duration of DSS administration, its concentration (anywhere between 1% and 5%) and molecular weight, the mouse strain to which DSS is given (eg, BALB/c and C57BL/6 mice are considered to be more susceptible strains), and the microbial environment of the treated mice (ie, specific pathogen-free, SPF vs GF mice).^8^ Depending on the combination of these factors, mice normally develop acute or chronic forms of colitis,^9^ but can even result in colitis-induced dysplastic lesions,^10^ although in the latter two cases, long-term administration with repeated cycles of DSS, or a combined pretreatment with a carcinogen, that is, azoxymethane (AOM), respectively, is required.^11^ The DSS model induces pronounced weight loss (approximately 5%-10% reduction by day 5 of administration), increased intestinal permeability (as shown by modulation of tight junction proteins after as little as 1 day of DSS administration), and altered stool consistency, leading to diarrhea, hematochezia, and in some cases, mortality.^12^ Acute DSS colitic mice display overt histological alterations, including mucin and goblet cell depletion, epithelial erosions associated with ulcerations, and infiltration of granulocytes into the lamina propria and submucosa.^6,9^ Notably, increased intestinal permeability also causes a dysbiotic state and contributes to the colitis phenotype.^13^ It is important to point out that the DSS colitis model also has limitations, including the fact that, differently from the human condition, T and B cells are not required for the development of colitis, making this model more suitable for the study of the innate versus adaptive arm of gut mucosal immunity for its contribution to the development of intestinal inflammation.^9^ Nonetheless, the timing, simplicity, and reproducibility of the DSS model make this one of the most widely used methods for the induction of colitis. However, the reliance on a chemical agent, the nature of the acute enterocyte-ischemic damage, as well as the lack of prominence of adaptive immune responses, limit the translational relevance of the DSS model to the human condition, particularly the chronic inflammatory state characteristic of UC patients. To this end, the use of alternative models of colitis that more closely resemble the pathology observed in patients with IBD is needed for pre-clinical *in vivo* investigation.

A widely used, genetically modified murine model of colitis is the interleukin-10 (IL-10)–deficient mouse, which was generated by Kühn et al. in 1993.^14^ These mice are characterized by an early onset of gut inflammation that occurs after weaning and develop histologic features similar to IBD. Colitis in IL-10 knockout (KO) mice results from aberrant T helper 1 (Th1) immune responses and consequent abundance of proinflammatory cytokine secretion from CD4^+^ T cells, particularly IL-12, IL-17, and interferon-gamma (IFN-γ). Colitis in IL-10-KO mice appears to be dependent on the gut microbiome since colitis is not observed under GF conditions, and treatment of SPF-raised IL-10-KO mice with either neomycin/metronidazole or ciprofloxacin prevents colitis for up to 12 weeks.^14^ Interestingly, the occurrence of colitis in IL-10-KO mice on a BALB/c background is reported to be 100% at 3 months, with an average disease severity score that is higher than that found in IL-10-KO mice on a C57BL/6 background. Specifically, 60% of BALB/c IL-10-KO mice develop transient endoscopic left-sided colitis, while 40% never show any endoscopic evidence of inflammatory lesions.^15^ The potential lack of persistent colitis, beyond the onset, could be perceived as a downside of this model. In addition, IL-10-KO mice in the same colony, and even littermates, are documented to develop non-uniform colitis, while other studies report different outcomes in IL-10-KO mice under SPF conditions in differing institutions/facilities, including histologically undetectable colitis^16^ or development of less severe colitis.^17^

Another model used to recapitulate IBD is multi-drug resistance 1 alpha (MDR1α) KO mice. MDR1α is a multi-drug resistance pump for the transport of small molecules across cell membranes. Multi-drug resistance 1 alpha KO mice that are housed under SPF conditions develop spontaneous colitis, with histological evidence of intestinal pathology present at approximately 12-15 weeks of age. The onset can be prevented with administration of antibiotics in drinking water, suggesting that colitis in these mice is dependent on microbiota; similarly, GF-raised MDR1α-KO mice also do not develop colitis. Multi-drug resistance 1 alpha KO mice show a severe thickening of the mucosa, with robust inflammatory cell infiltration into the lamina propria, as well as crypt abscesses and ulcerations extending throughout the colonic mucosa. The defect in MDR activity that leads to IBD resides in epithelial cells, indicating that a defect in barrier function is likely the triggering event of colitis. Dysregulation of immune responses is also part of the MDR1α-KO phenotype; *Ifng*, *Il6*, IL-1-β (*Il1b*), tumor necrosis factor (*Tnf*), C-C motif chemokine ligand 2 (*Ccl2*), *Ccl3*, *Ccl5*, and *Ccl11* are all increased in this model, in a similar manner to what it is observed in IBD patients.^18^

**Figure 1.**
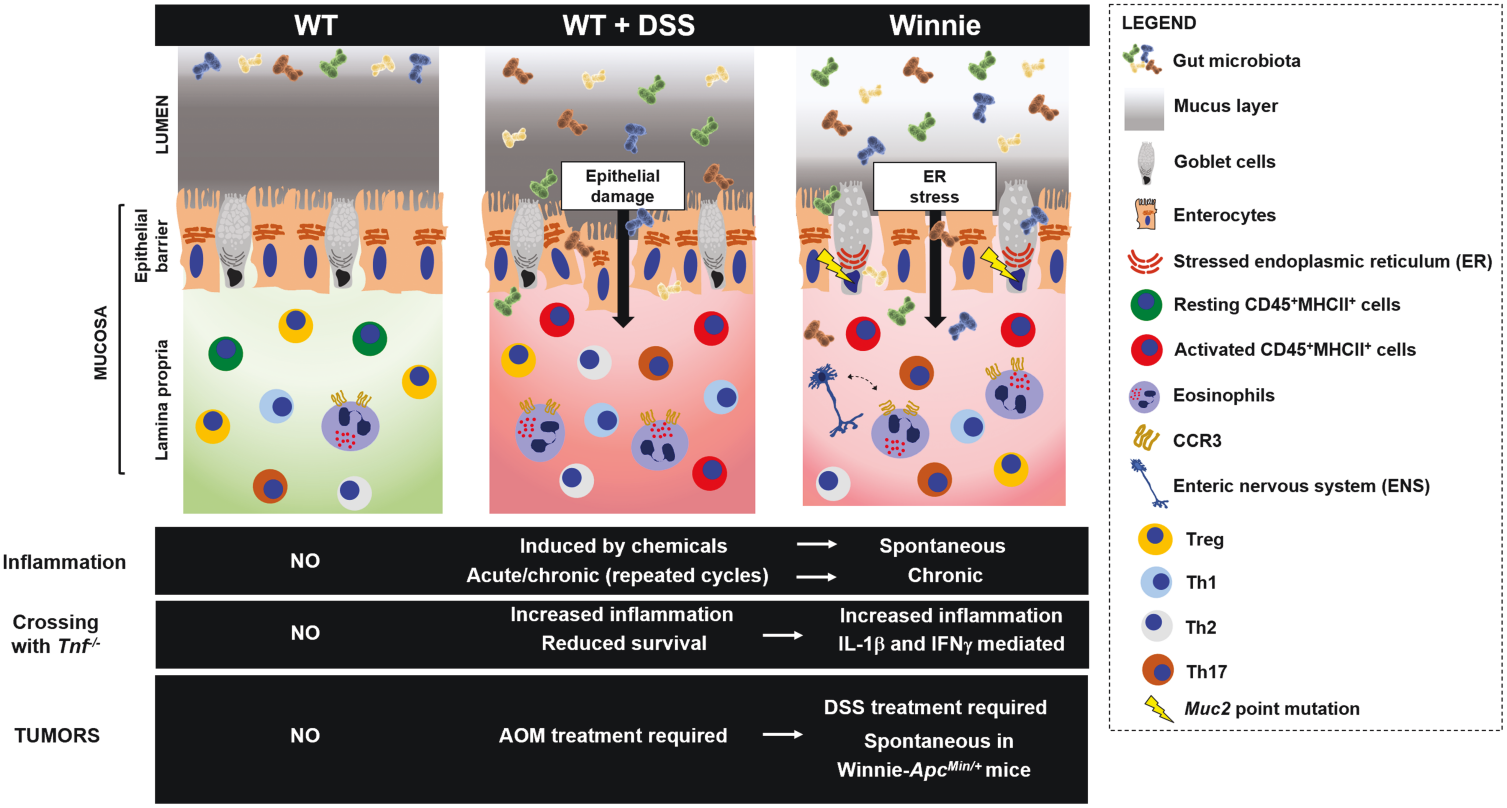
Winnie mice: A chronic and progressive model of ulcerative colitis and colitis-associated cancer. Intestinal homeostasis is a dynamic mechanism supported by the physical, chemical, and immunological characteristics of the host. Mucus secretion is fundamental to restraining bacterial presence within the intestinal lumen, with only occasional interaction with the epithelial barrier. Immune cells recruited into the lamina propria support immunological tolerance and Treg polarization. Acute inflammation mediated by dextran sodium sulfate (DSS) administration is caused by ischemic lesions of the epithelial barrier, abnormal bacteria translocation, and consequent inflammatory immune cell recruitment and activation. Mild, but chronic, intestinal inflammation caused by the *Muc2* mutation, such as that observed in Winnie mice, results in endoplasmic reticulum stress, leaky barrier, an inflammatory milieu conditioning immune cells towards T helper cells 17, C-C chemokine receptor type 3-mediated eosinophil recruitment, and pathologic eosinophil-enteric nervous system crosstalk. Notably, the absence of tumor necrosis factor exacerbates the intestinal pathology in both Winnie and DSS experimental colitis models. Abbreviations: AOM, azoxymethane; APC, adenomatous polyposis coli; IFN-γ, interferon-gamma; IL-1β, interleukin-1 beta; Muc2, mucin 2; Treg, T regulatory cells; WT, wild-type.

Considering that alterations of the intestinal mucus layer appear to be a main driver of the inflammatory state in UC, mouse models with aberrancies in mucin 2 (MUC2) have been developed.^19^ Specifically, mice deficient in *Muc2* are characterized by a loss of the mucus layer with pronounced invasion of intestinal bacteria and consequent inflammation, supporting the increased risk of colon cancer development^20,21^ (Figure 1). Similarly, mice harboring an alteration in *Muc2*, specifically a single missense genetic mutation, result in spontaneous colitis, with increased intestinal permeability and cytokine production in the distal part of the large intestine.^22^ This mutation was induced by injections of *N*-ethyl-*N*-nitrosourea (ENU) to C57BL/6 mice; colitis-affected mice were first out-crossed and subsequent litters then inter-crossed to map the mutation on chromosome 7, in the locus that contains *Muc2* and *Muc6*. Further investigation revealed that the mutagen randomly induced a missense mutation in *Muc2* (G9492A), resulting in substitution of cysteine with tyrosine at the N-terminal domain of the protein.

## Characterization of the Winnie Mouse Strain

Winnie mice were first described in 2008 by Heazlewood et al., demonstrating a mild spontaneous distal intestinal inflammation with chronic diarrhea that developed by 6 weeks of age in 100% of mice carrying the *Muc2* mutation.^22^ The gut pathology primarily occurred in the distal colon, showing crypt elongation, goblet cell loss, crypt abscesses, focal epithelial erosions, and neutrophilic infiltrates as classical signs of colitis.^22^ As in patients with UC, other typical features of the Winnie model are accumulation of the MUC2 precursor, endoplasmic reticulum (ER) stress in goblet cells, activation of the unfolded protein response (UPR), dysregulation of inflammatory gene expression, altered wound repair, and apoptosis. Notably, *MUC2* mutations are also common in UC patients, as recently reported in GWAS,^23^ further supporting the important role of MUC2 during the onset of UC, and validating Winnie mice as a relevant model of colitis.

The epithelial defect observed in Winnie mice induces a complex multi-cytokine-mediated colitis characterized by a Th1/Th2, and a prominent IL-23/Th17 response similar to that reported in UC patients.^22,24^ Crossing of Winnie with *Rag1*^−*/*−^ mice that lack T and B lymphocytes (ie, Winnie-*Rag1*^−*/*−^ mice, RaW) reveals a different role for innate and adaptive immune responses in this model.^24^ Specifically, while the former drives induction of the chronic inflammatory state in the distal colon, the latter supports its exacerbation, confirmed by the severe colitis reported in RaW mice receiving adoptively transferred naïve T cells from C57BL/6 mice.^24^

Chronic administration of DSS to Winnie mice results in a more severe phenotype. These mice have a shorter colon length and increased histological scores (ie, more severe colitis) compared to vehicle control-treated Winnie mice. Sustained chronic inflammation induces the formation of dysplastic crypts in the distal part of the colon of all DSS-treated Winnie mice. These adenomatous foci express increased Ki67, demonstrating increased proliferation at the base of the crypts.^25^ Moreover, β-catenin positivity is only detected in the membrane of dysplastic cells,^25^ a feature that is similar to UC-associated cancer, due to lack of genetic mutations in adenomatous polyposis coli (*APC*) or β-catenin.^26^ Dextran sodium sulfate-exposed Winnie mice have abundant C-X-C motif chemokine ligand 5 (CXCL5)-expressing mucosal leukocytes, possibly indicating progression from dysplasia to carcinoma.^27^ Furthermore, dysplastic epithelium in Winnie mice expresses genes related to apoptosis and cell proliferation, including *Myc*, caveolin 1 (*Cav1*), and *Trp53*, which are also often mutated and trigger colitis-associated cancer (CAC).^28^ As such, one advantage of this model is that a phenotype similar to CAC can be recapitulated, without using any carcinogens.

More recently, Winnie mice have been crossed with *Apc*^*Min/+*^ mice (ie, Winnie-*Apc*^*Min/+*^ mice) to demonstrate that a mild, but chronic, inflammatory state drives the onset and progression of tumoral lesions in mice that also carry a mutation (ie, *Apc*) common to individuals with a genetic predisposition to colorectal cancer (CRC).^29^ Importantly, dysplastic aberrant crypt foci (ACFs) already develop in 5-week-old Winnie-*Apc*^*Min/+*^ mice. Importantly, the Winnie-*Apc*^*Min/+*^ model has the advantage of not being induced chemically, such as the widely used AOM/DSS model,^11^ and develops colon-specific tumors, unlike the *Apc*^*Min/+*^ model that is characterized by a germline *Apc* mutation, resulting in polyps within the small intestine, but not in the colon.^30^

The Winnie-*Apc*^*Min/+*^ model has also been used to demonstrate the dual role of TNF in CAC by depleting *Tnf* from the established murine line. TNF deficiency was previously reported to increase DSS-induced colitis and reduce mice survival.^31^ While TNF promotes the onset of neoplastic lesions in the early stage of disease, it also induces their reduction during disease progression in TNF-deficient Winnie-*Apc*^*Min/+*^ mice.^32^ These data are extremely interesting considering conflicting reports regarding increased CRC risk, as well as other cancers, in IBD patients treated with monoclonal antibodies that block TNF, that is, Infliximab.^33,34^ In addition, the recent use of antibodies against checkpoint inhibitors results in a new form of colitis, that is, immune-related adverse event colitis (irAE-colitis). This new form of colitis appears to be improved by combination therapeutic strategies, based on concomitant use of anti-inflammatory biologics.^35,36^ Overall, these data underscore a complex and dynamic balance between inflammation and tolerance in mucosal tissues,^37^ with the concept ruling out one player often leading to an unexpected outcome. As such, relevant animal models remain a critical tool to develop precision-based treatment approaches for well-described, as well as emerging, inflammatory syndromes.

## Winnie Mice as a Reliable Pre-Clinical Model

Chronic ER stress plays a crucial role in colonic inflammation and is emerging as an area of increasing interest in the scientific community. Wilson et al. isolated goblet cells from Winnie mice and performed a proteomic analysis to find several pathways affected by ER stress.^38^ The most upregulated proteins found are those related to ER chaperon and UPR families, the same fate shared with mitochondrial chaperones; all of these aforementioned mechanisms are part of the cellular response to misfolded proteins and are a consequence of MUC2 aberrant production. Furthermore, ER stress induces a state of proteostasis in Winnie goblet cells, as mechanisms of transcription and translation are all downregulated; this is crucial as cells attempt to reduce the load on an already stressed ER. Some bioenergetic pathways related to fatty acid metabolism and oxidative phosphorylation are also upregulated in Winnie goblet cells, indicating a sign of increased oxidative stress, which is further corroborated by increased modulation of “redox-active center” and “peroxisome” functions. Among the stress relief proteins, coiled-coil-helix-coiled-coil-helix domain containing 2 (CHCHD2), a key survival protein, is upregulated in Winnie goblet cells and its protein localizes to the nuclei.^39^

Since Winnie mice represent a unique model of IBD induced by ER stress caused by MUC2 misfolding, Das et al. utilized this model to demonstrate, for the first time, that glucocorticoid administration is able to inhibit ER stress independent of nuclear factor-kappa beta (NF-κB) suppression.^40^ Glucocorticoids have been widely used to manage IBD as immunosuppressive agents; their efficacy is confirmed in Winnie mice as dexamethasone (DexM) inhibits the production of inflammatory cytokines, particularly from mesenteric lymph node (MLN)-derived T cells.^40^ Similarly, Hasnain et al. showed the importance of the anti-inflammatory cytokine, IL-10, in maintaining correct MUC2 folding; in support of this finding, administration of neutralizing anti-IL-10 antibodies exacerbates ER stress and pathologic signs of intestinal inflammation. These findings demonstrate a role for IL-10 in the correct folding and secretion of mucins within the intestinal mucosa, which is lost during chronic inflammation.^41^ Interestingly, administration of recombinant IL-20 is able to induce transient recovery in Winnie mice, but not in DSS-induced colitis; in particular, IL-20, which belongs to the IL-10 superfamily, reduces diarrhea and IL-6 production by decreasing the frequency of MLN-derived activated macrophages. Conversely, in DSS-treated mice, no significant changes are observed in the disease phenotype. Although IL-20 administration was not able to produce significant improvements in Winnie, the study by Moniruzzaman et al. underscores the advantages of using Winnie mice versus DSS colitic mice as a more suitable model of chronic intestinal inflammation.^42^

During chronic intestinal inflammation, inflammasome activation,^43^ and cytokine secretion^44^ are increased and thought to be responsible for the onset and flares that characterize the chronic relapsing nature of UC. In Winnie explants and purified immune cells from the intestinal lamina propria, both CD45^+^MHCII^+^ and CD45^+^MHCII^−^ cells secrete different types of cytokines at higher concentrations.^45^ When compared with wild-type (WT) mice, secretion of numerous cytokines and chemokines are significantly elevated (eg, IL-1ɑ, IL-1β, TNF, IL-4 IL-6, IL-12, IL-17, IL-18, IFN-γ, CCL3).^46,47^ A recurrent mediator shown to be important in the Winnie pathology is IL-1β, whose activation is a deleterious predictor of intestinal pathology; moreover, this cytokine is found more abundant in the colons of Winnie-*Tnf*^*−/−*^ mice, while their sera show greater levels of IFN-γ, IL-6, and IL-1ɑ. Notably, these findings are in line with what is observed in UC patients who do not respond to Infliximab therapy. These features are partially reversed upon Anakinra infusions.^48^ Similarly, Perera et al. demonstrated that inhibition of NLRP3 by MCC950 is able to decrease IL-1β release and caspase-1 activation.^46^

In general, these studies demonstrate the importance of using different murine models to understand various aspects of pharmacological treatments, particularly in the context of a multifactorial disease, such as UC. As previously discussed, Winnie mice are characterized by a Th17 profile, but the absence of T cells in the double mutant RaW model does not protect from colitis development, yet decreases disease progression.^24^ Winnie- *Il17a*^*−/−*^ mice have been generated to investigate the importance of IL-17 in disease onset and progression in Winnie mice. Surprisingly, the comparison between Winnie and Winnie-*Il17a*^*−/−*^ reveals minor differences. Treatment with monoclonal anti-IL-17 antibodies fails to ameliorate colitis in Winnie mice, confirming what is observed in the double mutant mice.^49^ Interestingly, encouraging results are obtained using monoclonal antibodies against IL-23, which acts upstream of IL-17A. IL-23 is essential for Th17 expansion and survival, but it is important to note that naïve T cells do not express the IL-23 receptor.^49^ Administration of anti-IL-23p19 antibodies is able to ameliorate established colitis in 12-week-old Winnie mice and reduce epithelial cell-mediated neutrophil infiltration; it is also able to decrease the amount of *Il1b* in the distal colon of these mice. In this context, results obtained using the Winnie model are more translationally relevant compared to what is observed using DSS colitic mice, due to the involvement of T and B cell immunity compared to the latter. Furthermore, Winnie can be crossed with *Rag1*^−*/*−^ mice to obtain RaW mice in order to transfer CD45R^hi^ cells, thus resulting in a T cell-focused colitis similar to what is described by Asseman et al.^50^

As supported by the recent literature, the gut–brain axis is fundamental in dictating intestinal health/disease states.^51,52^ Specifically, neuronal interactions with different gut mucosal immune populations (mainly mast cells, macrophages, and T- and B-cells) are reported to form a neuronal-immune axis that can be mainly reshaped by gut microbiota.^53–55^ Apart from the reported immune cell populations, the impact of eosinophil activity on the nervous system has gained attention in the context of IBD pathogenesis.^56,57^ Despite being only 5% of total cells within the circulation, eosinophils play a pivotal role in inflammatory cascades within the gastrointestinal tract; in fact, chronic intestinal inflammation also correlates with their accumulation in the areas of gut inflammation, as well as corresponds to the presence of nerve fibers.^58,59^ Eosinophil recruitment in the inflamed intestinal mucosa is a CCR3-mediated mechanism. In line with what is observed in chemically induced, chemokine-deficient mice, and various sublines of SAMP1/Yit mice, a spontaneous model of chronic ileitis,^60–62^ CCR3 blockade in Winnie mice has the ability to reduce eosinophil accumulation, both in the inflamed colon and within the circulation, thereby attenuating disease severity and morphological damage.^63^ The systemic effects also rely on a decrease in circulating eosinophil-associated cytokines and chemokines after targeting CCR3 receptors. However, the role of eosinophils in IBD and the interactions shared among eosinophil recruitment/activation and both the enteric and central nervous system (CNS)s are still largely unexplored. Thus, the Winnie model may represent an opportunity for understanding new aspects related to unconventional disease comorbidities in UC patients.

Similarities exist between the CNS and the enteric nervous system (ENS), which share many synaptic ultrastructure features. The latter is divided into myenteric and submucosal plexuses, based on their localization and are composed of thousands of small ganglia interconnected by neural fibers that can be negatively affected by IBD in regard to cell numbers and neuronal profiles.^64^ In turn, this can cause alterations in gastrointestinal motility associated with IBD symptoms that are usually reported by patients as chronic diarrhea and visceral hypersensitivity with frequent episodes of pain.^65,66^ Winnie mice show inherent features of a damaged ENS, including impairment of sensory innervation, abnormal intestinal transit, and altered colonic motility. Specifically, the rectal innervation of Winnie mice is compromised; this is especially true for mice with rectal prolapse,^67^ which is another typical feature of the Winnie model and it is usually associated with reduced rectal motor activity and anal sensitivity.^67^ While its occurrence is equal in both male and female Winnie mice, rectal prolapse has a higher incidence in female breeders after birthing.^67^ In addition, although it is a rare complication of UC,^68,69^ this condition primarily affects menopausal women,^70^ thus demonstrating the importance of Winnie as a unique and reliable model of IBD and intestinal inflammation-related pathologies.

## Role of the Gut Microbiome in Winnie Mice

Gut microbiota is among the key factors involved in the onset of chronic colitis. Several studies highlight the role of specific genera and species in the promotion of inflammation, while many other different species confer protection from exaggerated immune responses. The Winnie model closely resembles the human condition and its correlation with intestinal microbiota. Compared with same-mother WT littermates, it is observed that dysbiosis is already present in Winnie mice immediately after weaning, which progresses as mice age, worsening as the disease escalates from mild to chronic colitis. The crucial role of gut microbiota in driving intestinal inflammation becomes evident considering that its modulation is already reported during the early stage of disease. Four-week-old Winnie mice show an increased presence of *Verrucomicrobia* compared to their WT littermates; after 4 weeks, Winnie mice harbor an increased population of *Bacteroidetes* and fewer *Deferribacteres*. Chronic colitis induces a significant reduction in the phyla, *Proteobacteria* and *Deferribacteres*, compared with healthy littermate controls. At the genus level, Winnie mice show an increased abundance of *Bacteroides*, *Parabacteroides*, *Clostridium*, and *Paraprevotella*, while WT mice have more abundant *Blautia*, *Lactobacillus,* and *Mucispirillum*. Interestingly, chronic colitis and the *Muc2* mutation are also related to an increased abundance of *Akkermansia muciniphila* (*A. muciniphila*), several *Bacteroides* species, and *Lactobacillus taiwanensis*.^45^*Akkermansia muciniphila* abundance inversely correlates to several host inflammatory conditions, including IBD, obesity, and diabetes, and its therapeutic administration is reported to have protective effects.^71^ The abundance of *A. muciniphila* may, at least partially, be the result of a less tight mucus layer, favoring the overgrowth of mucus-degrading bacteria and is a unique characteristic of Winnie mice, perhaps representing a sub-group of UC patients. It is, therefore, thought that the *Muc2* mutation is responsible for the chronic colitis phenotype, as well as the early onset of dysbiosis. In addition, differentially represented bacterial populations are similar to what is found in patients with UC, as many of these genera are related to increased inflammation and exacerbation of immune stimulation.

Intestinal microbiota is a crucial component of inflammasome activation and it's hypothesized to trigger the development of dysplastic lesions in genetically predisposed mice. The aforementioned Winnie-*Apc*^*Min/+*^ mice, derived from crossing double heterozygote Winnie^*+/−*^-*Apc*^*Min/+*^ males with single heterozygous Winnie^*+/−*^ females, develop ACFs at 5 weeks of age, differently from age-matched parental controls, except for occasional ACFs in *Apc*^*Min/+*^ siblings. The occasional ACFs in *Apc*^*Min/+*^ are the result of a partial dysbiotic signature transferred from Winnie^*+/−*^ mothers chosen for the breeding strategy. Moreover, fecal transplantation of Winnie donor feces to *Apc*^*Min/+*^ recipients leads to an increased rate of ACF formation, suggesting that the dysbiotic signature in the gastrointestinal tract of Winnie breeders can be a transmittable risk factor driving the development of ACFs in resulting offspring.^29,72^

To reinforce the importance of the gut microbiota in supporting intestinal inflammation, Wang et al. demonstrated that Winnie mice housed under GF conditions show fewer signs of colitis compared to mice housed under conventional conditions.^73^ Germ-free-raised Winnie mice show a lower histological score and also fewer macroscopic signs of colitis, demonstrating that the *Muc2* mutation is not alone sufficient to cause the onset of colitis, but requires specific microbiota conditions. Nevertheless, this mutation is also responsible for perturbations that affect eubiotic microbiota and lead to chronic and progressive dysbiosis.^73^ However, this study was hampered by unforeseeable issues, as Winnie mice were transferred to a different facility; as such, the possibility exists that stress of the shipment might be responsible, at least partially, for the residual inflammation observed in GF-raised Winnie. These results suggest the importance of potential soluble mediators released by Winnie as a consequence of stress.

## Winnie Mice and Dysbiosis: A Model for Nutraceutical Formulation

Winnie mice are also a useful model to study the beneficial effects of probiotics and prebiotics, as these formulations possess a stable microbiota that does not change with age.^45^ Dietary intake is known to induce alterations in gut microbiome composition and several nutritional strategies may be important to ameliorate dysbiosis, potentially acting as adjuvants for conventional drugs.^74–76^ Polyphenols, in particular, are widely accepted as nutraceutical products able to suppress chronic inflammation by modulating the ability of intestinal immune cells to release inflammatory cytokines.^37,77^ Pujara et al. successfully used oral administration of β-lactoglobulin-encapsulated resveratrol for 2 weeks to improve body weight and reduce the disease activity index of Winnie mice.^78^ Mechanistically, one of the key cytokines responsible for the suppression of colitis is IL-10, which is also elevated in Winnie mice fed with a polyphenol-enriched metabolically engineered Bronze tomato line.^79^ The administration of Bronze tomatoes to Winnie breeding pairs results in alterations of the intestinal microbiome, in both mothers and litters, ultimately leading to a reduction in dysbiosis.^80^ Bronze tomato polyphenols are able to induce dramatic changes in Winnie microbiota^80^; genera like *Prevotellaceae_UCG-001* and *Lachnospiraceae_NK4A136-group*, known to promote production of short-chain fatty acids (SCFAs) production and anti-inflammatory abilities, are increased in mice that receive Bronze tomatoes before weaning and until sacrifice at 16 weeks of age. Similarly, 2 weeks of treatment to adult Winnie mice are enough to affect the intestinal microbiota and reduce the inflammatory milieu.^81^ Winnie microbiota and its associated metabolites are also responsible for the induction of an inflammatory response in Caco-2 intestinal epithelial cells and THP-1 macrophages; genes like *Ifng* and *Tnf* are upregulated by Winnie stool homogenates, while *Il10* is reduced. Moreover, Winnie stool increases the permeability of Caco-2 monolayers, measured by reduced transepithelial electrical resistance (TEER) and the expression of tight junction proteins.^82^

A probiotic/prebiotic formulation consisting of *Bacillus coagulans* and sugarcane fibers reduces the inflammatory burden at both the colonic and systemic levels in Winnie mice. This supplement reduces dysbiosis and improves the production of SCFAs by beneficial bacteria. *Oscillospira* and *Prevotella* are among the genera that benefit from changes in diet and increased SCFAs production; conversely, *Proteobacteria*, often associated with dysbiosis, are reduced, alongside *Desulfovibrio*.^83^ Furthermore, Winnie mice are susceptible to treatment with a cocktail of 5 antibiotics (1 g/L ampicillin, 0.2 g/L metronidazole, 1 g/L neomycin sulfate, 0.5 g/L vancomycin, and 0.1 g/L gentamicin); this treatment reduces mucosal inflammation through the selective depletion of pathogenic bacteria. Less inflammation is also confirmed with the analyses of cytokines, such as TNF and Il-1β, that are found to be reduced after antibiotic treatment, as well as decreased severity of colitis, in Winnie mice gavaged with the antibiotic mix.^84^

An innovative class of chemicals with interesting anti-microbial activity has also been tested using Winnie mice. The administration of short-chain quinones is still an understudied strategy to treat chronic intestinal inflammation, although several studies report their potent anti-inflammatory potentials.^85,86^ In Winnie mice, the administration of naphthoquinone (UTA77) results in reduced inflammatory responses, improved epithelial barrier integrity, and an overall decrease in the severity of colitis. Further investigation is required to determine whether UTA77 is effective in targeting intestinal dysbiosis of Winnie mice, or if the observed effects are dictated by the host response. Nonetheless, these results once again highlight the importance of a spontaneous and progressive model, like Winnie mice, and its use for pre-clinical studies of UC treatment.^87^

The Winnie model has also been used to investigate the possible use of thiopurines to suppress dysbiosis and modulate the relative percentage of harmful intestinal microbial species. Administration of thioguanine for 28 days affects populations of *Bacteroides thetaiotaomicron*, *Escherichia coli*, and *Enterococcusfaecalis*; these 3 species are increased by thioguanine and are responsible for its conversion to thioGMP, thioGDP, and thioGTP, which are supposed promoters of bacterial autophagy. This phenomenon is found to be less prominent in IBD, while its occurrence favors mucosal healing and reduces inflammation in the Winnie model.^88^

Microbiota of 8-month-old Winnie has also been proven to be a determinant in the onset of colon cancer when it is administered by gavage to antibiotic-treated *Apc*^*Min/+*^ recipients. *Akkermansia muciniphila* positively correlates, while *Lactobacillus intestinalis* negatively correlates to tumor formation, with the former species involved in tumor formation in UC patients, while the latter is often used as a probiotic. Other species, such as *Rikenella microfusus* and *Mucispirillum schaedleri*, show protective effects against tumor formation and negatively correlate with the development of cancer in Winnie/*Apc*^*Min/+*^ mice.^29,32,72^

Finally, Winnie mice present features associated with IBD that also affect other organs. Chronic intestinal inflammation and leaky barrier result in malabsorption, micronutrient deficiency, and consequently, bone mass and structural defects resembling human osteoporosis. Gut-enhanced permeability, dysbiosis, an imbalance between Th17/T regulatory cells (Tregs), and Vitamin D insufficiency are all connected with alveolar bone loss,^89^ long known to exist in patients with chronic inflammatory syndromes.^90^ Due to their peculiar characteristics, Winnie mice represent a perfect model to address the UC-osteoporosis axis,^91^ as well as nutritional strategies based on fortified nutraceuticals.^92^

## Conclusions

Winnie mice represent a reliable model of UC, due to their ability to develop a spontaneous, chronic, and progressive inflammation in the large intestine. Distinct from chemically or genetically induced colitis models, the inherent features of Winnie mice recapitulate several of the intricate interactions among immune, epithelial, neuronal cell populations, and colonic microbiota, thus resembling the complexity of the human pathology (Figure 1). Moreover, the fact that the Winnie phenotype is spontaneous, 100% penetrant, and that disease is already present in young (4-week-old) mice, presents an advantage compared to IL-10-KO and MDR1α-KO mice, whose features are a result of genetic manipulation and are often not observed in all mice. The Winnie model also represents a useful tool to investigate the extraintestinal manifestations related to IBD, providing another significant advantage over commonly used models of colitis. Furthermore, the chronic intestinal inflammation characterizing Winnie mice makes them suitable to address the effects of environmental or genetic triggers, as well as develop precision medicine-based approaches for predisposed individuals. In addition, the combination of studying the *Muc2* mutation with other cancer-related mutations has already paved the way for dissecting the mechanism(s) of inflammation and colorectal cancer. In particular, Winnie-*Apc*^*Min/+*^ mice represent a valid model for the early onset of CRC (in patients younger than 50 years).^93^ In fact, the incidence of early-onset CRC is predicted to increase sharply in the next few years^94,95^; thus, a model for disease prevention and treatment will become even more important in the near future.
